# Elevated free interleukin-18 associated with severity and mortality in prospective cohort study of 206 hospitalised COVID-19 patients

**DOI:** 10.1186/s40635-022-00488-x

**Published:** 2023-02-24

**Authors:** Syed M. T. Nasser, Anas A. Rana, Rainer Doffinger, Andreas Kafizas, Tauseef A. Khan, Shuaib Nasser

**Affiliations:** 1 Intensive Care Department, Surrey and Sussex NHS Foundation Trust, Redhill, UK; 2 Present Address: Intensive Care Department, Royal Surrey County Hospital, Egerton Road, Guildford, GU2 7XX UK; 3 Centre for Computational Biology, Birmingham University, Birmingham, UK; 4 Department of Clinical Biochemistry and Immunology, Cambridge University Hospitals NHS Trust, Cambridge, UK; 5 The Grantham Institute for Climate Change and the Environment, Imperial College London, South Kensington, London, UK; 6 Department of Chemistry, Molecular Science Research Hub, Imperial College London, White City, London, UK; 7 Department of Nutritional Sciences, Faculty of Medicine, University of Toronto, Toronto, Canada; 8 Department of Allergy, Cambridge University Hospitals NHS Trust, Cambridge, UK

**Keywords:** Cytokines, Inflammasomes, Pneumonia, Viral, Autoimmunity, Coronavirus

## Abstract

**Background:**

Divergence between deterioration to life-threatening COVID-19 or clinical improvement occurs for most within the first 14 days of symptoms. Life-threatening COVID-19 shares clinical similarities with Macrophage Activation Syndrome, which can be driven by elevated Free Interleukin-18 (IL-18) due to failure of negative-feedback release of IL-18 binding protein (IL-18bp). We, therefore, designed a prospective, longitudinal cohort study to examine IL-18 negative-feedback control in relation to COVID-19 severity and mortality from symptom day 15 onwards.

**Methods:**

662 blood samples, matched to time from symptom onset, from 206 COVID-19 patients were analysed by enzyme-linked immunosorbent assay for IL-18 and IL-18bp, enabling calculation of free IL-18 (fIL-18) using the updated dissociation constant (K_d_) of 0.05 nmol. Adjusted multivariate regression analysis was used to assess the relationship between highest fIL-18 and outcome measures of COVID-19 severity and mortality. Re-calculated fIL-18 values from a previously studied healthy cohort are also presented.

**Results:**

Range of fIL-18 in COVID-19 cohort was 10.05–1157.7 pg/ml. Up to symptom day 14, mean fIL-18 levels increased in all patients. Levels in survivors declined thereafter, but remained elevated in non-survivors. Adjusted regression analysis from symptom day 15 onwards showed a 100 mmHg decrease in PaO_2_/FiO_2_ (primary outcome) for each 37.7 pg/ml increase in highest fIL-18 (*p* < 0.03). Per 50 pg/ml increase in highest fIL-18, adjusted logistic regression gave an odds-ratio (OR) for crude 60-day mortality of 1.41 (1.1–2.0) (*p* < 0.03), and an OR for death with hypoxaemic respiratory failure of 1.90 [1.3–3.1] (*p* < 0.01). Highest fIL-18 was associated also with organ failure in patients with hypoxaemic respiratory failure, with an increase of 63.67 pg/ml for every additional organ supported (*p* < 0.01).

**Conclusions:**

Elevated free IL-18 levels from symptom day 15 onwards are associated with COVID-19 severity and mortality. ISRCTN: #13450549; registration date: 30/12/2020.

**Supplementary Information:**

The online version contains supplementary material available at 10.1186/s40635-022-00488-x.

## Background

The Coronavirus disease 2019 (COVID-19) pandemic continues to cause significant disruption across the world. Despite intense efforts, consensus has not yet been reached on the pathogenic processes that lead to life-threatening disease.

Investigators have highlighted parallels between life-threatening COVID-19 infection and secondary Hemophagocytic Lymphohistiocytosis (HLH) also known as “Macrophage Activation Syndrome” (MAS), on the basis of genetic [1], clinical [2], histopathological [3], cytological [4] and immunological [5] similarities. MAS is a heterogenous syndrome encompassing different aetiologies; however, one type of MAS, NLR family CARD domain-containing protein 4 (NLRC-4) mutation-related, was discovered in 2018 to be pathologically driven [6] by loss of negative-feedback control of interleukin-18 (IL-18) due to failure of its binding protein (IL-18bp) production, resulting in elevated levels of the biologically active “free” IL-18 (fIL-18) component. This study, therefore, aims to investigate whether dysfunctional negative-feedback of IL-18 is associated with life-threatening COVID-19 disease.

IL-18 is the end-product of inflammasome activation. The inflammasome [7] comprises of pattern-recognition receptors (PRRs), stimulated by “pathogen-associated molecular patterns” (PAMPS; e.g., bacterial toll-like receptor agonists) or “damage-associated molecular patterns” (DAMPS, e.g., signals of tissue injury). Activated PRRs organise into multi-protein cytosolic structures called inflammasomes, found across cell types. The final common pathway of different inflammasomes is activation of the enzyme caspase-1, which cleaves pro-IL-18 to its active, free form. fIL-18 potently augments interferon-gamma (IFN-gamma) production, but is also capable, in coordination with interleukin-2 (IL-2), in regulating a type-2 immune response [8]. IL-18 additionally stimulates interleukin-6 (IL-6) and Granulocyte–macrophage colony-stimulating factor (GM-CSF) [9], blockade of which have yielded therapeutics for severe COVID-19 [10, 11]. IL-18 induces the release of its own binding protein (IL-18bp), which binds to it in a 1:1 ratio, at a higher affinity than the IL-18 alpha chain (IL-18Rα), the receptor ligand for mature fIL-18 [12]. IL-18bp, therefore, acts as a soluble decoy receptor, with high endogenous inhibition of IL-18 activity, in a negative feedback circuit. As such, on the assumption that the dissociation constant remains constant in different disease states, measurement of fIL-18 is a direct way to assess the integrity of this negative feedback circuit [40]; increased production of IL-18 without commensurate release of the binding protein will result in increased fIL-18. Measurement of total IL-18, therefore, without calculation of the “free”, biologically active component, can produce misleading results [13].

Research early in the pandemic showed that the multi-system inflammatory deterioration of life-threatening COVID-19, noted for its similarities to HLH, occurs between days 9 and 14 (interquartile range) of symptom onset [14]. This fits with findings that Computerised Tomography (CT)-based resolution of inflammatory lung changes in COVID-19 occur from symptom day 15 onwards [15]. Both these sources indicate symptom day 15 as a key timepoint differentiating those clinically improving, and those deteriorating to life-threatening disease. Therefore, to investigate whether loss of IL-18 negative feedback control is associated with life-threatening disease in COVID-19, we designed an observational, prospective cohort study looking at IL-18 negative feedback control from symptom day 15 onwards, in hospitalised patients testing positive with COVID-19. This was achieved through measurement of total IL-18 and IL-18bp and calculation of the fIL-18 component. This was undertaken in 206 COVID-19 positive patients during hospital admission, matched to day from symptom onset. Adjusted multivariate regression analyses were then used to quantify the strength of association between fIL-18 and disease severity and mortality outcomes, from symptom day 15 onwards.

## Methods

### Study setting and patient selection

The study was conducted at East Surrey Hospital, UK, with the period of recruitment running from the 9th October 2020 to 9th January 2021.

Inclusion criteria to the cohort were: SARS-COV_2_ polymerase-chain reaction (PCR) swab-positive result; age > than 18 years; inpatient admission. All patients were recruited to the study within 24 h of positive PCR result. This included patients who contracted COVID-19 while admitted to hospital for other medical or surgical reasons. There were no exclusion criteria; hospitalised patients at all levels of disease severity, including asymptomatic patients, were enrolled. If enrolled patients were discharged with COVID-19 but re-admitted, they were not re-enrolled, but continued under the same enrolment. During the recruitment period 272 patients met the inclusion criteria. As per standard of care at the time of recruitment, all patients enrolled in this study, who required supplemental oxygen, were initiated on a daily course of Dexamethasone 6 mg for 10 days.

### Blood sample collection

Standard Operating Procedures to collect blood samples prospectively throughout the course of hospital admission for cytokine analysis, involved the following. First, Ethylenediaminetetraacetic acid (EDTA) plasma and serum separator tubes used to collect blood samples for clinical purposes, were taken, with serum samples being allowed to clot for up to 30 min after harvesting and centrifuged immediately thereafter. Centrifugation of all samples was conducted at between 1500 and 2000 g at room temperature (17–21 °C) for 15 min. 0.5–1 ml supernatant was then aliquoted using a sterile, multichannel pipette and stored in a 2 ml sterile Eppendorf ^®^ tube. These were then stored at − 70 °C within 8 h of harvesting from patients. Aliquots were matched to “day from symptom onset” in 219 patients for whom the date of symptom onset was recorded in clinical notes.

### ELISA testing and fIL-18 calculation

**Fig. 1:**
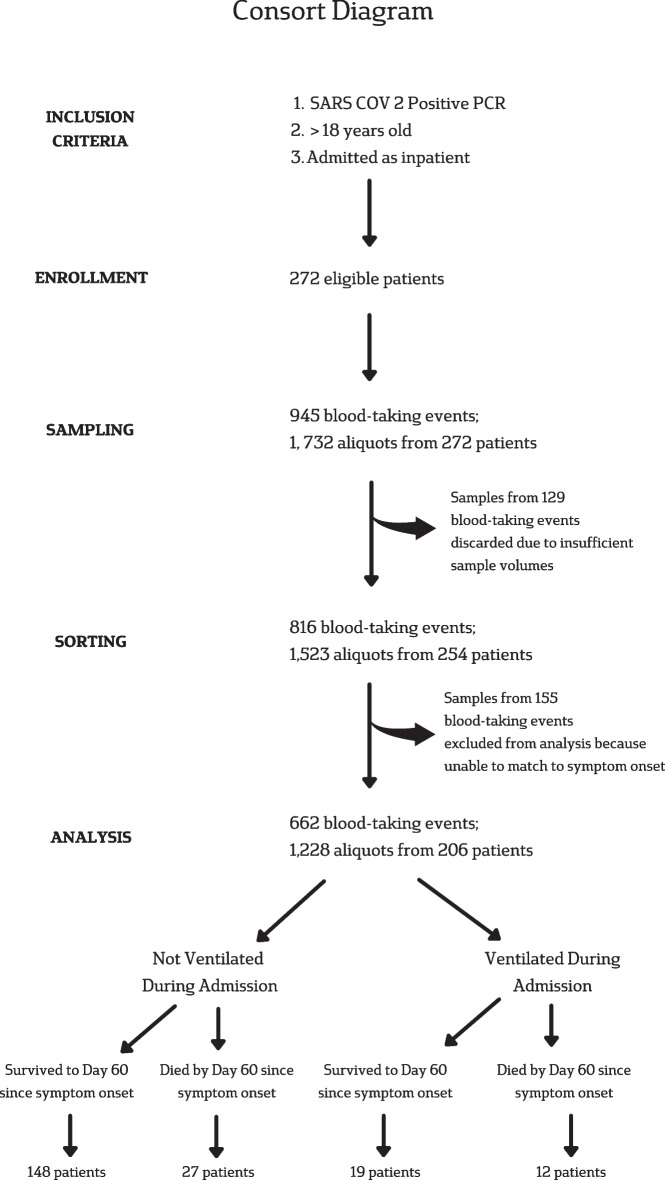
Consort diagram. Inclusion criteria enabled enrolment of 272 eligible patients, from whom 1732 aliquots of serum and Ethylenediamine tetraacetic acid (EDTA) plasma and serum blood samples were obtained. Discarding of samples due to insufficient volumes for enzyme-linked immunosorbent assay (ELISA) left 1523 aliquots, of which, 1228 could be matched to day from symptom onset, from 206 patients

EDTA plasma and serum samples were thawed at room temperature before enzyme-linked immunosorbent assay (ELISA) analysis. Total IL-18 (Human total IL-18/IL-1F4 Quantikine ELISA kit, R&D Systems, 5 Minneapolis, MN, USA; sensitivity: 5.15 pg/ml; coefficient of variance: 8.33%) and IL-18 Binding Protein (IL-18bp) (Human IL-18bpa Quantikine ELISA kit, R&D Systems, Minneapolis, MN, USA; sensitivity: 7.52 pg/ml; coefficient of variance: 8.90%) were measured. IL-18 and IL-18bp analysis were always conducted from the same EDTA or serum sample. All ELISA procedures were undertaken at Cambridge University Hospital’s Cytokine Laboratory, Cambridge, UK, in accordance with the manufacturer’s protocol. The flow of participants from enrolment to analysis is presented in a Consort Diagram (Fig. 1) and the basis for exclusion of 66 patients, resulting in final analysis of 1228 aliquots from 206 patients. No samples measured were below the quantification limit.

Calculation of fIL-18 levels were determined from Total IL-18 and IL-18bp using the established 1:1 stoichiometry and dissociation constant (K_d_) of 0.05 nM in a law of mass-action calculation as per recent evidence [16] (see Additional file 1 for calculation steps). IL-18 and IL-18bp provided from historical data [17] in 442 non-COVID-19, healthy male volunteers were re-analysed (K_d_ = 0.05 nM) to present baseline population fIL-18 levels. Results with fIL-18 levels calculated using the historical K_d_ = 0.4 nM [18] for purposes of comparison to past studies, are presented in the Supplementary Materials.

### Patient characteristics and outcome data collection

Patient characteristics and outcome data collection were recorded from clinical notes for the final 206 patients whose samples were used for statistical analysis.

### Patient characteristics

Demographic and patient characteristic data recorded for all patients were: age; race; days since symptom onset at enrollment; sex; admission diagnosis; incidence of comorbidities: hypertension, diabetes, congestive cardiac failure, chronic kidney disease, and chronic lung disease; medications; number of blood samples taken per patient. Patient characteristics are presented in Table 1.

**Table 1:** Baseline characteristics of 206 patients by 60-day mortality outcome

Characteristic	Survival to day 60 from symptom onset (n = 165)	Died by day 60 from symptom onset (n = 41)	Significance testing
Age			
Mean, year	69.8 [59–82]	77.4 [69–88]	***p* < 0.01**
Distribution, %			
< 70 years	44	29	–
70–79 years	21	22	
≥ 80 years	34	49	
Sex, %			
Male	53	63	–
Female	47	37	
Race, %^¶^			
White	64	54	–
Minority: Asian	5	7	
Minority: Black	2	2	
Other/undisclosed	28	37	
Admission diagnosis (%)			
COVID-19	84	85	–
Other^±^	16	15	
Median no. of days since symptom onset [IQR]*	7 [4–10]	6 [3–10]	–
Median no. of blood samples taken per patient [IQR]*	2 [1–4]	4 [2–6]	***p* < 0.05**
No. of patients discharged prior to symptom day 15 (%)	89 (54%)	2 (4.8%)	**–**
No. of patients died prior to symptom day 15 (%)	–	14 (34%)	
Sample type analysed for IL-18 parameters (%)			
Serum	89	91	–
EDTA plasma	11	9	
Artificial mechanical ventilation status at any point during admission			
Not ventilated	145 (88)	29 (71)	*X*^2^ (1, *N* = 206) = 9.10 ***p* < 0.01**
Received artificial mechanical ventilation	20 (12)	12 (29)	
Previous coexisting disease—no. (%)^‡^			
Any of the below listed diseases	114 (69)	39 (95)	*X*^2^ (1, *N* = 153) = 32.5 ***p* < 0.001**
Diabetes	37 (22.4)	11 (26.8)	–
Hypertension	76 (46.0)	24 (58.5)	–
Congestive cardiac failure	16 (9.7)	5 (12.2)	–
Chronic kidney disease	59 (35.8)	23 (56.1)	*X*^2^ (1, *N* = 82) = 4.48 ***p* < 0.05**
Chronic lung disease	20 (12.1)	7 (17.1)	–

Results that reach the significance threshold are bolded

*IQR refers to “interquartile range”

^¶^Race or ethnic group was recorded in the patient’s electronic health record

^±^Other admission diagnoses include the following (*n*): orthopaedic injury secondary to fall (15); stroke (4); fall without orthopaedic injury (3); pulmonary embolus (2); abdominal pain (2); confusion (2); decompensated congestive cardiac failure (2); sepsis (2); diarrhea (1); discitis (1); acute limb ischaemia (1); acute kidney injury (1); cellulitis (1); facial injury (1); subdural haemorrhage (1); trifascicular block (1); supraventricular tachycardia (1); small bowel obstruction (1); infective arthritis (1); hyperglycaemia (1); heart valve disorder (1)

^‡^Co-morbidities were determined in the following manner: diabetes, hypertension, congestive cardiac failure and chronic lung disease were determined from diagnosis in patient notes; chronic kidney disease was determined as a recorded estimated Glomerular Filtration Rate < 90 in the year preceding admission or diagnosis in medical notes at the time of admission

### Outcome data

Outcome data recorded from blood sampling included: sample type (EDTA plasma or serum); lymphocyte count; neutrophil count; C-reactive protein; ferritin. Outcome data relating to respiratory parameters of the patient at the time of blood sampling were also recorded, including: fraction of inspired oxygen (FiO_2_); method of oxygen delivery; partial pressure of oxygen (PaO_2_) from arterial blood gas sampling or, if not available, derived from oxygen saturations (SaO_2_) as described ([SaO_2_/FiO_2_]–29.6/1.09) [19] and PaO_2_/FiO_2_ ratio (PFR). Outcome data relating to organ dependency parameters included use of vasopressor, renal replacement therapy and artificial mechanical ventilation. Outcome data relating to 60-day mortality was recorded from electronic clinical notes, including patient Summary Care Record, which is updated contemporaneously.

### Statistical analysis

All statistical analysis was carried out using *R*.

### Longitudinal shape of the data

All IL-18 parameters (fIL-18, total IL-18 and IL-18bp) as well as all outcome data listed above, were categorised into bins by time from symptom-onset: days 1–4, 5–9, 10–14, 15–19, 20–24 and 25–29. There was no previous data to inform this categorisation; these time frames were chosen as a balance between granularity and numbers of samples per bin for meaningful comparison while enabling analysis of samples from symptom day 15 onwards. Longitudinal data of all IL-18 parameters and outcome data are presented in Table 2.

**Fig. 2:**
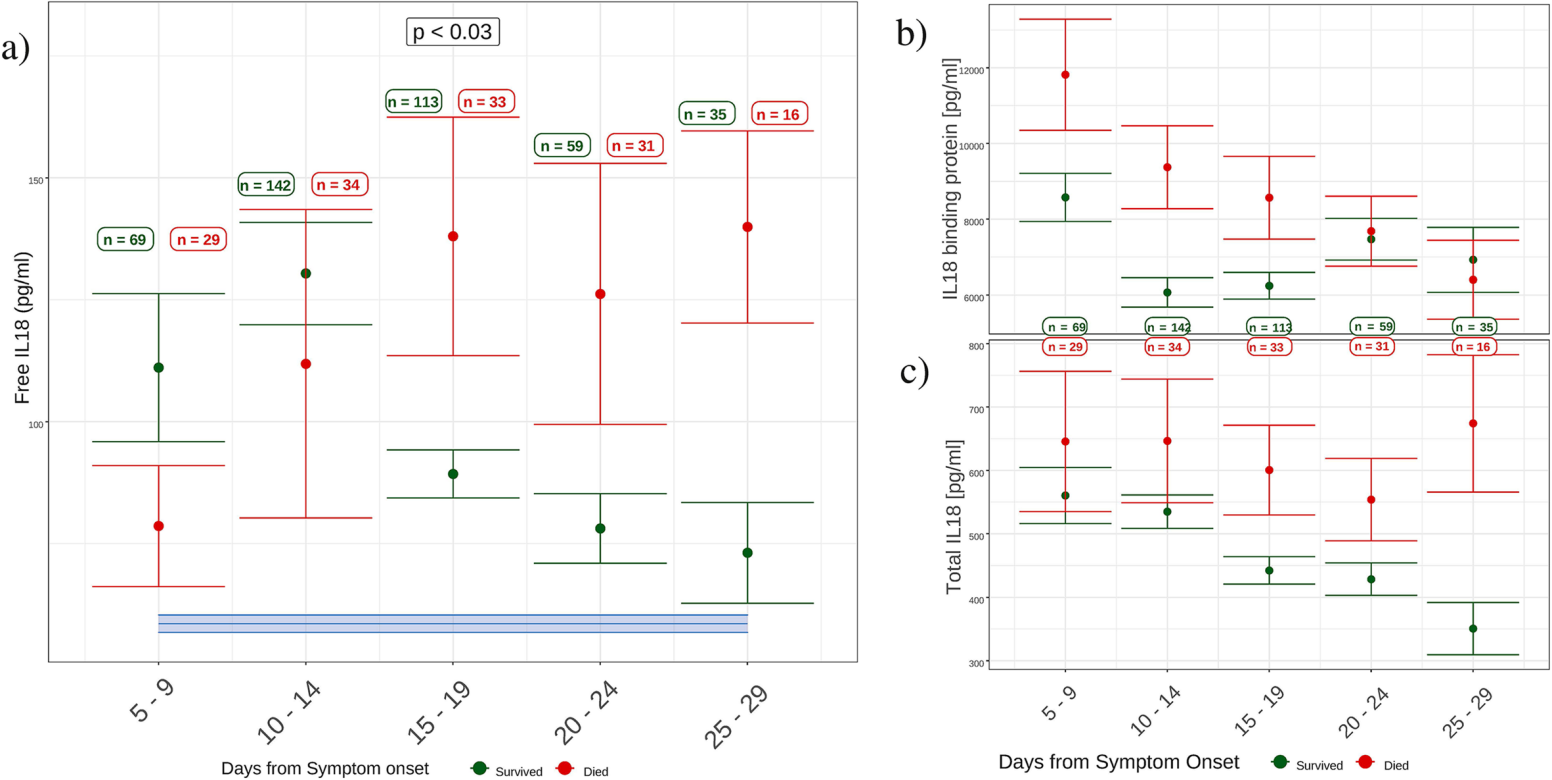
IL-18 parameter profiles by day from symptom onset (K_d_ = 0.05 nM). **a** Levels of fIL-18 increase between symptom days 1–14 days in 206 COVID-19 positive patients from baseline. From symptom day 15 onwards, fIL-18 levels diverge between survivors and non-survivors (days 15–19: 89.27 pg/ml [survived] vs. 138.00 pg/ml [died]; *p* < 0.03). Mean fIL-18 level in 442 healthy, male volunteers is 58.6 pg/ml (blue bar). **b** Levels of IL-18 binding protein (IL-18bp) after symptom day 15 overlap between survivors and non-survivors. **c** Total IL-18 increases in 60-day non-survivors from symptom day 15 onwards. Error bars represent standard error of the mean. *Survivors (black): 499 samples from 165 patients. Non-survivors (red): 162 samples from 41 patients*

Box plots of fIL-18, Total IL-18 and IL-18bp values by time from symptom onset, separated by 60-day mortality outcome, were constructed to present the shape of the data (Fig. 2). Separation by 60-day mortality was chosen as a more intuitive way of understanding this longitudinal exploratory analysis, though PaO_2_/FiO_2_ ratio (PFR) remained the primary outcome in the main statistical, regression analysis. Unpaired student’s *T* test was used at days 15–19 on fIL-18 values, to verify whether the divergence in clinical condition from symptom day 15 onwards, referred to earlier, is reflected in fIL-18 levels. We further constructed the same box plots in the sub-group of patients requiring artificial mechanical ventilation at any point during admission (see Additional file 1).

Conclusions were not drawn from *T* tests conducted on IL-18 parameter profiles, constructed only to show shape of the data, rather, conclusions were only drawn from the results of adjusted multivariate regression models.

### Regression analyses

Since IL-18 parameter profiles, described above, took each blood sample as an individual data point, without considering that some patients had more blood samples than others, adjusted multivariate regression analysis, accounting for patient-related confounders, could only be conducted after selecting one fIL-18 value per patient. We chose “highest fIL-18” value per patient, from symptom day 15 onwards, to ensure capture of the greatest degree of dissociation between IL-18 and its binding protein. Use of mean or median values were avoided, since they would have resulted in comparison of individual values to averaged values, due to differences in the number of blood samples obtained per patient.

All regression analyses were adjusted for by age; sex; comorbidities: diabetes, hypertension, congestive cardiac failure, chronic kidney disease and chronic lung disease; admission diagnosis (COVID-19 or other) and sample tube collection type (EDTA plasma or Serum). All regression analyses are presented in Table 3.

#### Disease severity

**Fig. 3:**
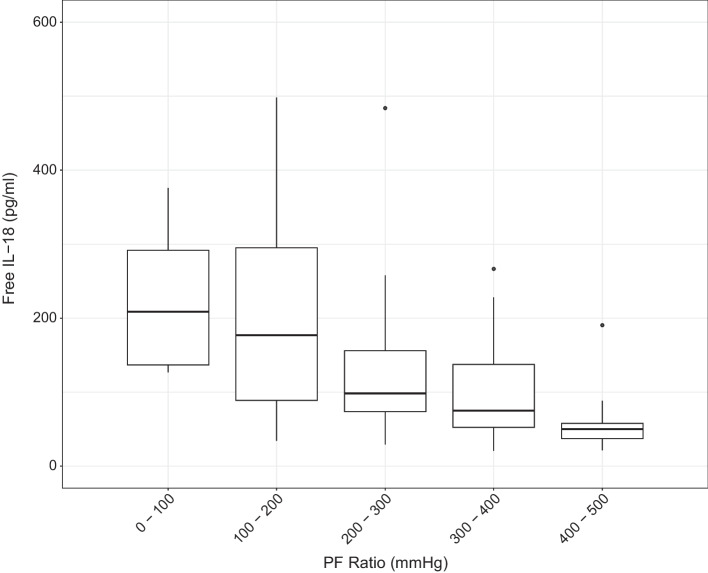
PaO_2_/FiO_2_ ratio (PFR) vs. Highest Free IL-18 (fIL-18) from symptom day 15 onwards. Highest fIL-18 per patient (*n* = 97) from symptom day 15 onwards shows an inverse, statistically significant relationship with concurrent PFR (primary outcome), divided into bins as per the Berlin Criteria for acute respiratory distress syndrome (ARDS) severity categories, and extended above 300 mmHg. Multivariate adjusted linear regression shows an increase of 37.7 pg/ml in highest fIL-18 for each decrease in PFR by 100 mmHg (13.3 kPa) (*p* < 0.03)

Linear regression was conducted with highest fIL-18 from symptom day 15 onwards, against: PaO_2_/FiO_2_ ratio (PFR) as the primary outcome (Fig. 3); CRP, Lymphocyte count as a percentage of white blood cells, neutrophil/lymphocyte ratio (NLR); concurrent number of organs supported, as defined by: vasopressor use, artificial mechanical ventilation, and renal replacement therapy (RRT). Comparison to ferritin levels could not be conducted due to insufficient data. Linear regression against NLR and number of organs supported, was repeated in a sub-population that excluded patients who had not died with hypoxaemic respiratory failure, as defined below.

Logistic regression with highest fIL-18 from symptom day 15 onwards as the covariate, was conducted against concurrent organ support of any kind (out of vasopressor use, artificial mechanical ventilation, and renal replacement therapy) and concurrent artificial mechanical ventilation only.

#### Mortality analysis

Adjusted logistic regression analysis was performed with highest fIL-18 as the covariate and crude 60-day mortality, as measured from onset of symptoms, as the outcome. Adjusted logistic regression analysis was then repeated with 60-day mortality, as measured from onset of symptoms, with hypoxaemic respiratory failure, as the outcome. This was performed to help exclude patients dying from causes unrelated to COVID-19, but having contracted COVID-19 as inpatients. Criteria for exclusion of patients from the “hypoxaemic respiratory failure” mortality group was: no PFR < 300 mmHg in recorded observations within 24 h of death. This was conducted blinded to patient fIL-18 values, resulting in the exclusion of 4 out of 22 patients.

## Results

### Baseline characteristics

Of the 206 patients analysed for IL-18 parameters, 165 survived to day 60 from symptom onset, and 41 died. Mean age between the two groups was significantly different (*p* < 0.01), though the distribution of ages was not found to be significantly different. There was a greater proportion of males in the mortality group (63% vs. 53%). These findings are in keeping with previous reports [20] that age and male gender are risk factors for mortality with COVID-19 infection. The distribution of race was not significantly different between the two groups. Median number of days from symptom onset were similar between survivors and non-survivors (7 days vs. 6 days). Median number of blood samples taken per patient were significantly different (*p* < 0.05) with non-survivors having a higher median number of samples (2 samples vs. 4 samples). Of the fully enrolled cohort, 54% of 165 60-day survivors were discharged prior to symptom day 15 and thus not included in the regression analysis, being deemed to no longer have active disease, and thus at risk of introducing bias into the regression analysis. Of 41 60-day non-survivors, prior to symptom day 15, 2 patients were discharged and 14 patients died. The 2 discharged non-survivors were deemed inappropriate for blood sampling due to their palliative status. Survivors showed less than half the rate of artificial mechanical ventilation as compared to non-survivors (12% vs. 29%) (*p* < 0.01), as would be expected. Having any comorbidity was significantly associated with mortality as compared to no comorbidity (*p* < 0.001) and of the comorbidities, only incidence of chronic kidney disease was significantly different in non-survivors than survivors, being higher in the former group (*p* < 0.05).

### Longitudinal shape of the data

Detailed results of all data, separated by 60-day mortality outcome, longitudinally collected, are presented in Table 2. These include data related to IL-18 parameters, biochemical parameters and organ support parameters.

**Table 2:** Longitudinal data on IL-18, biochemical and clinical parameters

IL-18 parameters	60-day mortality outcome	Days from symptom onset									
		5–9		10–14		15–19		20–24		25–29	
		Mean	Standard error	Mean	Standard error	Mean	Standard error	Mean	Standard error	Mean	Standard error
Total IL-18 (pg/ml)	Survived	560.33	44.27	534.84	26.32	442.43	21.48	428.64	25.39	350.72	41.13
	Died	645.59	110.60	646.47	96.10	600.49	70.78	553.83	65.02	674.19	108.40
IL-18 binding protein (pg/ml)	Survived	8574.96	635.56	6067.95	385.68	6243.67	347.08	7469.31	550.48	6927.66	856.63
	Died	11,818.55	1469.41	9373.12	1094.42	8567.37	1092.87	7683.42	924.45	6400.98	1039.35
Free IL-18 (pg/ml)	Survived	111.10	15.10	130.37	10.40	89.27	4.89	78.09	7.14	73.11	10.17
	Died	78.60	12.39	111.87	31.16	138.00	24.45	126.20	26.75	139.92	19 .69
Biochemical parameters											
C-reactive protein (mg/L)	Survived	59.17	6.35	52.48	5.01	38.76	5.07	66.31	12.02	56.74	12.11
	Died	76.16	10.63	93.41	14.73	84.67	13.74	78.13	16.13	58.73	11.54
Ferritin (ng/ml)	Survived	1060.67	286.63	1180.55	160.51	899.17	150.04	685.94	130.93	696.5	315.5
	Died	764.5	138.5	1157.33	226.2	1161.92	147.42	897.5	100.01	1159.56	140.82
Lymphocyte (% WCC)	Survived	14.21	1.62	12.14	1.1	18.41	6.68	11.96	1.01	13.22	1.27
	Died	13.92	4.1	8.93	2.67	17.34	4.53	16.21	4.21	19.46	7.04
Neutrophil/lymphocyte ratio (NLR)	Survived	10.23	1.18	12.5	0.92	11.19	0.89	9.6	0.8	8.79	1.33
	Died	14.82	2.31	20.43	2.39	20.29	3	14.24	1.72	10.87	1.71
Clinical parameters											
PaO_2_/FiO_2_ ratio (PFR)	Survived	314.4	13.2	286.42	8.9	278.48	10.6	285.43	13.5	296.77	18
	Died	270.87	23.72	223.13	21.87	210.73	20.13	180.22	20.9	159.53	30.77
Peak inspiratory pressure* (cmH_2_0)	Survived	29.75	1.65	28.63	1.05	23.08	0.98	23.45	1.48	11.33	1.69
	Died	29.2	2.13	23.78	1.77	24	2.14	24.92	1.18	29.23	1.67
Positive end expiratory pressure* (cmH_2_O)	Survived	7.5	2.63	6.07	1.16	7.94	0.79	7.55	0.822	7.33	0.42
	Died	12	1.22	10.44	0.73	9.75	0.92	10.58	0.36	10.54	0.94

*These parameters were measured only in the sub-group of those admitted to intensive care

Figure 2a shows the shape of the data in relation to fIL-18 levels. It shows how mean fIL-18 levels increased in survivors and non-survivors between symptom day 1 and symptom days 10–14. From symptom day 15 onwards, we see mean fIL-18 divergence between survivors and non-survivors (days 15–19: 89.3 pg/ml [survived] vs. 138 pg/ml [died]; *p* < 0.03). fIL-18 levels in survivors between days 15 and 29 from symptom onset, trend towards the level seen in healthy volunteers, represented by the blue bar. Profiles of IL-18bp and Total IL-18 levels, Fig. 2b and (c), respectively, show that the fIL-18 divergence after day 15 is due to increased production of IL-18 without a commensurate increase in the levels of IL-18bp.

### Regression analyses

Results of all regression analyses can be seen in Table 3.

**Table 3:** Regression analysis for primary and secondary outcomes from symptom day 15 onwards

Outcome K_d_ = 0.05 nM	Highest free IL-18 response coefficient (standard error)	Odds ratio per 50 pg/ml increase in highest free IL-18 (95% confidence interval)	Significance
Primary outcome			
PaO_2_/FiO_2_ Ratio (mmHg)	– 0.377 (0.111)		***p* < 0.03**
Secondary outcomes			
Mortality			
Death at 60 days from symptom onset (crude mortality)		1.41 (1.1–2.0)	***p* < 0.03**
Death at 60 days from symptom onset (hypoxaemic respiratory failure)^✸^		1.90 (1.3–3.1)	***p* < 0.01**
Organ-support dependency			
Any organ support^✦^		1.66 (1.1–2.9)	***p* < 0.05**
Artificial mechanical ventilation only		1.66 (1.00–2.9)	*p* = 0.05
Per additional organ supported^✦^	50.72 (23.0)		***p* < 0.05**
Per additional organ supported (hypoxaemic respiratory failure)^✦✸^	63.67 (23.3)		***p* < 0.01**
Biochemical inflammatory markers			
C-reactive protein (CRP)	0.065 (0.29)		*p* > 0.5
Lymphocyte (as % of white blood cells)	− 118.4 (109)		*p* > 0.2
Neutrophil/lymphocyte ratio	2.67 (1.3)		*p* = 0.05
Neutrophil/lymphocyte ratio (hypoxaemic respiratory failure)^✸^	3.54 (1.4)		***p* < 0.03**

Results that reach the significance threshold are bolded

^✸^The “hypoxaemic respiratory failure group” excludes 4 patients whose calculated PaO2/FiO2 ratio from values recorded in the last 24 h of life did not fall below 300 mmHg at any time. The group is used to exclude those who died from causes other than hypoxaemic respiratory failure

^✦^“Per Additional Organ supported” refers to each new organ supported, out of: vasopressor support; artificial mechanical ventilation; renal replacement therapy

### Primary outcome: PaO_2_/FiO_2_ ratio (PFR)

Highest fIL-18 from symptom day 15 onwards was significantly inversely associated with concurrent PFR. Adjusted linear regression demonstrates that for every increase in highest fIL-18 by 0.377 pg/ml, PFR decreases by 1 mmHg, or to put another way, for every increase in highest per patient fIL-18 by 37.7 pg/ml, PFR decreases by 100 mmHg (*p* < 0.03). Figure 3 depicts the relationship between PFR, separated into bins of 100 mmHg as per the Berlin criteria [21] of ARDS, and highest per patient fIL-18 from symptom day 15 onwards. No parameters were significant confounders.

### Secondary outcomes

#### Mortality

Adjusted logistic regression gave an odds ratio (OR) of crude 60-day mortality of 1.41 (1.1–2.0) for each 50 pg/ml increase in highest fIL-18 from symptom day 15 onwards (*p* < 0.03). When the outcome group is narrowed to those who died by day 60 with hypoxaemic respiratory failure, adjusted logistic regression gave an OR of 1.90 (1.3–3.1) for each 50 pg/ml increase in highest fIL-18 from symptom day 15 onwards (*p* < 0.01). No parameters were significant confounders with either regression analysis.

#### Organ failure

Adjusted logistic regression showed a relationship between highest fIL-18 from symptom day 15 onwards, and artificial mechanical ventilation status that was on the cusp of significance (*p* = 0.05). Taking *any* organ support (vasopressor support, artificial mechanical ventilation *or* renal replacement therapy) as the outcome, gave a significant OR of 1.66 (1.1–2.9; *p* < 0.05). For every additional organ supported out of vasopressors, artificial mechanical ventilation or renal replacement therapy, highest fIL-18 increased by 50.7 pg/ml (*p* < 0.05). When the same linear regression analysis was conducted, excluding those who did not die with hypoxaemic respiratory failure, adjusted linear regression gave an increase of highest fIL-18 of 63.67 pg/ml (*p* < 0.01) for each additional organ supported.

#### CRP, NLR and lymphocyte (%), ferritin

Adjusted linear regression of highest fIL-18 against concurrent CRP, NLR and Lymphocyte count (as percentage of white blood cells) from symptom day 15 onwards showed only NLR as having a significant association. For each increase in NLR by 1 in patients with hypoxaemic respiratory failure, highest fIL-18 increases by 3.54 (*p* < 0.03). In the full patient cohort, an increase in NLR by 1 increases highest fIL-18 by 2.67 (*p* = 0.05). Not enough patients had measured ferritin levels for adequate regression analysis.

## Discussion

We found that dysfunction of IL-18 negative feedback control is associated with disease severity and death in COVID-19 positive patients from symptom day 15 onwards. From symptom day 15 onwards, for every 37.7 pg/ml (K_d_ = 0.05 nM) increase in highest per patient fIL-18, PFR (primary outcome) decreased by 100 mmHg (13.3 kPa) (*p* < 0.03). For each 50 pg/ml increase in highest fIL-18 per patient from symptom day 15 onwards, the adjusted OR for crude 60-day mortality was 1.41 (1.1–2.0] (*p* < 0.03). When the outcome was limited to those who died with hypoxaemic respiratory failure, the OR increased to 1.9 (1.3–3.1); *p* < 0.01. For each increase in highest fIL-18 by 50 pg/ml, the OR for requiring any organ support, defined as vasopressor therapy, artificial mechanical ventilation or renal replacement therapy, was 1.66 (1.1–2.9); *p* < 0.05. For each additional organ supported, fIL-18 increased by 50.7 pg/ml (*p* < 0.05) and when only patients who died with hypoxaemic respiratory failure were analysed, this increased to 63.67 pg/ml (*p* < 0.01).

This study adds to the literature in two significant ways. First, this is the first report to characterise *free* IL-18 levels in COVID-19 throughout the course of the illness; previous studies characterised either only the total IL-18 component [22, 23] which does not reflect the biologically active interleukin and may hide the true picture of inflammasome activation [13] or only analysed free IL-18 levels at admission. The latter resulted in missing trends that occur from the point of clinical deterioration in the second week of the illness and onwards. Second, this is one of the few studies to undertake interleukin analysis in the context of the disease course, through careful correlation to the first day of symptom onset.

The population under study were hospitalized adults who tested positive for SARS-CoV-2 on PCR. The baseline features of the population relating to age and gender distribution were in keeping with other studies [24]. Ethnic differences between the mortality groups were not significantly different, but not representative of the region of the hospital, with minority groups being under-represented. It is possible that the “undisclosed” ethnicity category contained a larger proportion of ethnic minorities. There was no difference in median number of days from symptom onset, at the time of enrolment, between mortality groups, indicating that non-survivors do not present later to hospital than survivors. The mortality rate of patients not-ventilated (16%) and ventilated patients (37.5%) was higher than that described elsewhere (11.5% and 33%, respectively) [24]; however, this may have been due to the higher median age of participants in our study. The distribution of comorbidities was as expected between the mortality outcomes, though of note, only the difference in chronic kidney disease reached the significance threshold; hypertension incidence did not. This appears to be due to a lower incidence of hypertension (58.5%) in our non-survivors compared to that described elsewhere (67.6%) [24]. During inpatient stay, non-survivors had significantly more blood samples taken per patient, as expected, with patients in intensive care being bled daily, and having a higher mortality rate than patients not admitted to intensive care. While daily dexamethasone was included in the standard of care by the time of enrolment, IL-6 blockade had not yet been included. Thus, the fIL-18 profile observed in this study is in the context of steroid-based immunosuppression.

Two inflammasome types, NLRP3 and NLRC4, have classically been the main focus of study. IL-18 production from the NLRP3 inflammasome, an intracellular sensor of anti-microbial signals found mainly in macrophages, and which activates in response to the pathogenically stimulated ASC protein scaffold, shows how IL-18 can be detrimental to clinical outcomes along two axes: level and persistence of elevation. In models of sepsis, for example, injection of a low or moderate dose of lipopolysaccharide (LPS) induces a moderate rise in IL-18 levels that enhances anti-bacterial host defenses, while injection with high doses results in sustained, high levels of IL-18, that impair host antibacterial defenses [25]. In models of lung infection, avian influenzae H5N1 and H7N9, which contain a PB1–F2 protein, persistently activate NLRP3, resulting in persistently elevated levels of IL-18, inducing IFN-gamma, and a subsequent cytokine storm [26, 27] in a manner reminiscent to severe acute respiratory syndrome (SARS) [28]. This profile of a sustained, elevated IL-18 level associated with poor clinical outcomes is in keeping with our findings that persistently elevated fIL-18 levels from symptom day 15 onwards are significantly associated with disease severity and 60-day mortality, in COVID-19.

While NLRC4 is also activated by pathogenic signals, specifically flagellin and components of the type III secretion system [29], it is also capable of activating caspase-1 and producing IL-18 independently of the ASC scaffold and thus, unlike NLRP3, is not dependent on pathogenic stimuli. Thus, NLRC4 mutations can result in overwhelming production of IL-18, as in systemic juvenile idiopathic arthritis (sJIA) [30] and adult onset still’s disease [31], in which NLRC4 mutations drive IL-18 production into the nanogram range, due to uninhibited production.

Hemophagocytic lymphohistiocytosis (HLH), a syndrome that bears key similarities to life-threatening COVID-19 [1–5], is characterised by phagocytosis in bone marrow and other tissues, of haemoglobin, white blood cells and platelets by histiocytes, such as macrophages, under excess stimulation by IFN-gamma. The aetiology of excessive IL-18 production, and subsequent excess IFN-gamma stimulation, driving macrophage activation, differs by HLH type. For example, familial and secondary HLH, the latter also known as MAS, include macrophage activation due to lytic failure, by cytotoxic T-cells, of IFN-gamma producing antigen-presenting cells, due to intrinsic T-cell mutations [32]. MAS additionally is used to describe excessive release of fIL-18 due to uncontrolled inflammasome activation, due to NLRC4 mutations [33], again driving elevated IFN-gamma levels and macrophage activation. The third and final type, known as CpG-induced MAS, involves relentless antigenic stimulation of the NLRP3 inflammasome, through activation of toll-like receptor 9 (TLR9), which recognises DNA rich in unmethylated CpG–DNA motifs from bacterial or viral DNA, again driving elevated fIL-18 levels, and subsequent elevated IFN-gamma levels [34].

This third pathway of CpG-induced MAS through constitutive NLRP3 activation, may be the central pathomechanism of COVID-19. Early functional exhaustion of innate immunity, so crucial in early antigen-control, is seen in fatal COVID-19 [35]. Work by Waggoner et al. has shown the essential role of Natural Killer (NK) cells in modulating CD4 + T Cells to *prevent* such functional exhaustion [36]. As NK cell function and number are impaired with age [37] and in those with metabolic syndrome conditions [38] we would expect to see greater functional exhaustion of lymphocytes in these groups, unchecked viral spread, and repeated inflammasome stimulation, driving CpG-induced MAS, with fatal outcomes.

Both Weiss et al. [6] and Girard–Guyonvarc’h [34] et al. have shown the essential role of IL-18bp in silencing IL-18 activity in MAS and CpG-induced MAS, respectively. Our findings demonstrate that elevated fIL-18 after symptom day 15 is driven by increased production of IL-18 without a commensurate increase in IL-18bp (Fig. 2). Why adequate levels of IL-18bp are not released from symptom day 15 onwards under its usual homeostatic mechanism is unclear. Neutralising auto-antibodies to IFN-alpha, seen in life-threatening COVID-19 [39] may explain the elevated levels of fIL-18; IFN-alpha both diminishes IL-18 production from macrophages and is an important inducer of IL-18bp [40]. Interestingly, the PB1–F2 protein in H5N1 and H7N9, cited earlier, which drives a cytokine storm through excessive IL-18 production from mass activation of NLRP3 inflammasome [27], also inhibits IFN-alpha production [41]. Persistently elevated fIL-18 in severe COVID-19 may underlie the finding that elevated IFN-gamma after day 10 of symptoms is independently associated with death [42]; IFN-gamma potently activates macrophages [43] and macrophage-mediated destruction of lung architecture via infiltration of extra pulmonary tissue is a hallmark of fatal COVID-19 [44].

Weaknesses of this study include, first, the inherent limitations of being a single-centre, prospective observational study. Recognition of this early in study design was attempted to be mitigated through selection of a site which serves a racially diverse population. A second weakness relates to the use of PF ratios derived from SaO_2_ when PaO_2_ values were not available, albeit through a validated mathematical model. Right-shift in the haemoglobin dissociation curve in critically ill patients may have resulted in under-estimation of the PFR in the critically ill cohort, potentially under-estimating the slope of the association between highest fIL-18 and PFR from symptom day 15 onwards. This is unlikely to have played a significant role in introducing bias, however, since PFR was calculated from measured PaO_2_ values directly, in all critically ill patients. A third weakness relates to the incompleteness of our data on ferritin measurement. Hyperferritinaemia forms a part of the H-score used to diagnose MAS, and is particularly elevated with NLRC4 mutation-driven MAS. Though the ferritin levels seen in this study are certainly elevated (Table 2), the averaged values per time-bin do not reach the required threshold for contributing to the H-score (> 2000 ng/ml). This may be due to incomplete data collection, dexamethasone-mediated suppression, or simply, because life-threatening COVID-19 may not conform to all the diagnostic features of MAS as currently formulated, despite its similar clinical features and the association of elevated fIL-18 with disease severity and mortality. Finally, only four patients in the cohort received continuous veno-venous haemodiafiltration (CVVHDF) in our cohort, of whom, two died and two survived. Biologically active, mature IL-18 is 18 kilodaltons (kDa) [53], while the theoretical lower limit pore size of CVVHDF for convection is approximately 30–35 kDa [54]. A systematic review of extracorporeal cytokine removal reveals little data in relation to IL-18 removal, concluding that hybrid techniques such as CVVHDF generally result in low levels of cytokine clearance [55]. This may be due to their hydration shell which results in behavior as larger molecules would.

Areas of further research include, first, validating these results in a separate cohort. Second, comparing fIL-18 levels in patients with COVID-19 against other conditions, which may help clarify conflicting results [45, 46], though of note, these cited studies did not analyse the *free* IL-18 portion. In addition, concurrent analysis of IL-6, though not within the scope of our research question, could be an avenue of further research. Since IL-18 stimulates IL-6 release [47], it is unlikely IL-6 blockade would attenuate the fIL-18 profile; current understanding is that the IL-1β/IL-6/CRP and IL-18/ferritin inflammatory axes are separate [48], supported by the lack of association between fIL-18 and CRP in our study. Finally, though we focused our regression analyses on the period from symptom day 15 onwards on the basis of our research question, our longitudinal data indicates that non-survivors have higher IL-18bp early in the disease course (days 5–9). High baseline IL-18bp, as seen in metabolic syndrome conditions [49], may prevent a sufficient rise in fIL-18 necessary to facilitate a strong Th1 response for early antigen-control, resulting in antigen escape from symptom day 15 onwards, and persistent inflammasome-mediated IL-18 release. This is an area requiring further research.

This study demonstrates the potential utility of fIL-18 as a biomarker of disease, from symptom day 15 onwards in patients with COVID-19. While causation cannot be established in this observational study, our findings provide hypothesis-generating evidence for modulation of IL-18 from symptom day 15 onwards in patients with COVID-19. Our finding that for every 37.7 pg/ml (K_d_ = 0.05 nM) increase in highest fIL-18, PFR declines by 100 mmHg after symptom day 15 (Fig. 3), provides both a time frame and a theoretical approach for fIL-18 blockade based on the degree of hypoxaemic respiratory failure. Potential drug candidates for such an intervention include Tadekinig Alfa (AB2Bio), a recombinant human interleukin-18 binding protein, having shown efficacy in conditions with elevated fIL-18, such as MAS [50] and sJIA [30], and caspase-1 inhibitor, Belnacasan (Roivant), currently in Phase 2 trials for COVID-19 [51], having shown efficacy in reducing pulmonary inflammation in animal models [52].

## Conclusions

We report that failure of negative feedback control of IL-18, resulting in elevated free IL-18 from symptom day 15 onwards in patients hospitalised with COVID-19 infection, is associated significantly with disease severity, as determined by PaO2/FiO2 ratio, organ support dependency and 60-day mortality. Our results support the hypothesis that life-threatening COVID-19 may be MAS-like, and may benefit from IL-18 modulation from symptom day 15 onwards.

## Supplementary Information

**Additional file 1.** Supplementary Methods. Supplementary Results.

## Data Availability

The data sets used and/or analysed during the current study are available from the corresponding author on reasonable request.

## References

[1] Kaser A (2020). Genetic risk of severe COVID-19. N Engl J Med.

[2] Opoka-Winiarska V, Grywalska E, Roliński J (2020). Could hemophagocytic lymphohistiocytosis be the core issue of severe COVID-19 cases?. BMC Med.

[3] Prieto-Pérez L, Fortes J, Soto C, Vidal-González Á, Alonso-Riaño M, Lafarga M, Cortti MJ, Lazaro-Garcia A, Pérez-Tanoira R, Trascasa Á, Antonio A (2020). Histiocytic hyperplasia with hemophagocytosis and acute alveolar damage in COVID-19 infection. Mod Pathol.

[4] Weiskopf D, Schmitz KS, Raadsen MP, Grifoni A, Okba NM, Endeman H, van den Akker JP, Molenkamp R, Koopmans MP, van Gorp EC, Haagmans BL (2020). Phenotype and kinetics of SARS-CoV-2–specific T cells in COVID-19 patients with acute respiratory distress syndrome. Sci Immunol..

[5] Attwell L, Zaw T, McCormick J, Marks J, McCarthy H (2021). Haemophagocytic lymphohistiocytosis after ChAdOx1 nCoV–19 vaccination. J Clin Pathol.

[6] Weiss ES, Girard-Guyonvarc’h C, Holzinger D, de Jesus AA, Tariq Z, Picarsic J, Schiffrin EJ, Foell D, Grom AA, Ammann S, Ehl S (2018). Interleukin-18 diagnostically distinguishes and pathogenically promotes human and murine macrophage activation syndrome. Blood.

[7] Zheng D, Liwinski T, Elinav E (2020). Inflammasome activation and regulation: toward a better understanding of complex mechanisms. Cell Discovery.

[8] Nakanishi K, Yoshimoto T, Tsutsui H, Okamura H (2001). Interleukin-18 regulates both Th1 and Th2 responses. Annu Rev Immunol.

[9] Ogura T, Ueda H, Hosohara K, Tsuji R, Nagata Y, Kashiwamura SI, Okamura H (2001). Interleukin-18 stimulates hematopoietic cytokine and growth factor formation and augments circulating granulocytes in mice. Blood.

[10] Gordon AC, Mouncey PR, Al-Beidh F, Rowan KM, Nichol AD, Arabi YM, Annane D, Beane A, Berry LR, Bhimani Z, Bonten MJ (2021). Interleukin–6 receptor antagonists in critically ill patients with COVID-19. N Engl J Med.

[11] Temesgen Z, Burger CD, Baker J, Polk C, Libertin CR, Kelley CF, Marconi VC, Orenstein R, Catterson VM, Aronstein WS, Durrant C (2021). Lenzilumab in hospitalised patients with COVID-19 pneumonia (LIVE–AIR): a phase 3, randomised, placebo–controlled trial. Lancet Respir Med.

[12] Dinarello C, Novick D, Kim S, Kaplanski G (2013). Interleukin-18 and IL-18 binding protein. Front Immunol.

[13] Michels M, de Mast Q, Netea MG, Joosten LA, Dinarello CA, Rudiman PI, Sinarta S, Wisaksana R, Alisjahbana B, van der Ven AJ (2015). Normal free interleukin-18 (IL-18) plasma levels in dengue virus infection and the need to measure both total IL-18 and IL-18 binding protein levels. Clin Vaccine Immunol.

[14] Chen SL, Feng HY, Xu H, Huang SS, Sun JF, Zhou L, He JL, Song WL, Wang RJ, Li X, Fang M (2020) Patterns of deterioration in moderate patients with COVID-19 from Jan 2020 to Mar 2020: a multi–center, retrospective cohort study in China. Front Med 839 10.3389/fmed.2020.567296 PMC7744800 33344469

[15] Pan F, Ye T, Sun P, Gui S, Liang B, Li L, Zheng D, Wang J, Hesketh RL, Yang L, Zheng C (2020) Time course of lung changes on chest CT during recovery from 2019 novel coronavirus (COVID-19) pneumonia. Radiology 10.1148/radiol.2020200370 PMC7233367 32053470

[16] Girard C, Rech J, Brown M, Allali D, Roux-Lombard P, Spertini F, Schiffrin EJ, Schett G, Manger B, Bas S, Del Val G (2016). Elevated serum levels of free interleukin-18 in adult–onset Still’s disease. Rheumatology.

[17] Thompson SR, Novick D, Stock CJ, Sanders J, Brull D, Cooper J, Woo P, Miller G, Rubinstein M, Humphries SE (2007). Free Interleukin (IL)-18 levels, and the impact of IL18 and IL18BP genetic variation, in CHD patients and healthy men. Arterioscler Thromb Vasc Biol.

[18] Kim SH, Eisenstein M, Reznikov L, Fantuzzi G, Novick D, Rubinstein M, Dinarello CA (2000). Structural requirements of six naturally occurring isoforms of the IL-18 binding protein to inhibit IL-18. Proc Natl Acad Sci.

[19] Jusi RL, Limpin E, Bayot R, De Guia T, Ayuyao F (2015). Determination of critical threshold value of SPO2/FiO2 ratio in the diagnosis of acute lung injury. Philippine Heart Center J..

[20] Jordan RE, Adab P, Cheng K (2020). COVID-19: risk factors for severe disease and death. BMJ.

[21] Force AD, Ranieri VM, Rubenfeld GD, Thompson BT, Ferguson N, Caldwell E, Fan E, Camporota L, Slutsky AS (2012). Acute respiratory distress syndrome. JAMA.

[22] Satış H, Özger HS, Yıldız PA, Hızel K, Gulbahar Ö, Erbaş G, Aygencel G, Tunccan OG, Öztürk MA, Dizbay M, Tufan A (2021). Prognostic value of interleukin-18 and its association with other inflammatory markers and disease severity in COVID-19. Cytokine.

[23] Tjan LH, Furukawa K, Nagano T, Kiriu T, Nishimura M, Arii J, Hino Y, Iwata S, Nishimura Y, Mori Y (2021). Early differences in cytokine production by severity of coronavirus disease 2019. J Infect Dis.

[24] Nicholson CJ, Wooster L, Sigurslid HH, Li RH, Jiang W, Tian W, Cardenas CL, Malhotra R (2021). Estimating risk of mechanical ventilation and in–hospital mortality among adult COVID-19 patients admitted to Mass General Brigham: the VICE and DICE scores. EClinicalMedicine.

[25] Joshi VD, Kalvakolanu DV, Hasday JD, Hebel RJ, Cross AS (2002). IL-18 levels and the outcome of innate immune response to lipopolysaccharide: importance of a positive feedback loop with caspase–1 in IL-18 expression. J Immunol.

[26] Pinkerton JW, Kim RY, Robertson AA, Hirota JA, Wood LG, Knight DA, Cooper MA, O’Neill LA, Horvat JC, Hansbro PM (2017). Inflammasomes in the lung. Mol Immunol.

[27] McAuley JL, Tate MD, MacKenzie-Kludas CJ, Pinar A, Zeng W, Stutz A, Latz E, Brown LE, Mansell A (2013). Activation of the NLRP3 inflammasome by IAV virulence protein PB1–F2 contributes to severe pathophysiology and disease. PLoS Pathog.

[28] Huang KJ, Su IJ, Theron M, Wu YC, Lai SK, Liu CC, Lei HY (2005). An interferon-γ-related cytokine storm in SARS patients. J Med Virol.

[29] Sasaki Y, Otsuka K, Arimochi H, Tsukumo SI, Yasutomo K (2020) Distinct roles of IL–1β and IL-18 in NLRC4–induced autoinflammation. Front Immunol 2604 10.3389/fimmu.2020.591713 PMC7592392 33178225

[30] Yasin S, Solomon K, Canna SW, Girard-Guyonvarc’h C, Gabay C, Schiffrin E, Sleight A, Grom AA, Schulert GS (2020). IL-18 as therapeutic target in a patient with resistant systemic juvenile idiopathic arthritis and recurrent macrophage activation syndrome. Rheumatology.

[31] Gabay C, Fautrel B, Rech J, Spertini F, Feist E, Kötter I, Hachulla E, Morel J, Schaeverbeke T, Hamidou MA, Martin T (2018). Open–label, multicentre, dose–escalating phase II clinical trial on the safety and efficacy of tadekinig alfa (IL-18BP) in adult–onset Still’s disease. Ann Rheum Dis.

[32] Schulert GS, Cron RQ (2020). The genetics of macrophage activation syndrome. Genes Immun.

[33] Canna SW, de Jesus AA, Gouni S, Brooks SR, Marrero B, Liu Y, DiMattia MA, Zaal KJ, Sanchez GA, Kim H, Chapelle D (2014). An activating NLRC4 inflammasome mutation causes autoinflammation with recurrent macrophage activation syndrome. Nat Genet.

[34] Girard-Guyonvarc’h C, Palomo J, Martin P, Rodriguez E, Troccaz S, Palmer G, Gabay C (2018). Unopposed IL-18 signaling leads to severe TLR9–induced macrophage activation syndrome in mice. Blood.

[35] Zheng HY, Zhang M, Yang CX, Zhang N, Wang XC, Yang XP, Dong XQ, Zheng YT (2020). Elevated exhaustion levels and reduced functional diversity of T cells in peripheral blood may predict severe progression in COVID-19 patients. Cell Mol Immunol.

[36] Waggoner SN, Cornberg M, Selin LK, Welsh RM (2012). Natural killer cells act as rheostats modulating antiviral T cells. Nature.

[37] Ventura MT, Casciaro M, Gangemi S, Buquicchio R (2017). Immunosenescence in aging: between immune cells depletion and cytokines up–regulation. Clin Mol Allergy.

[38] Bähr I, Jahn J, Zipprich A, Pahlow I, Spielmann J, Kielstein H (2018). Impaired natural killer cell subset phenotypes in human obesity. Immunol Res.

[39] Bastard P, Rosen LB, Zhang Q, Michailidis E, Hoffmann HH, Zhang Y, Dorgham K, Philippot Q, Rosain J, Béziat V, Manry J (2020). Autoantibodies against type I IFNs in patients with life–threatening COVID-19. Science.

[40] Kaser A, Novick D, Rubinstein M, Siegmund B, Enrich B, Koch RO, Vogel W, Kim SH, Dinarello CA, Tilg H (2002). Interferon-α induces interleukin-18 binding protein in chronic hepatitis C patients. Clin Exp Immunol.

[41] Varga ZT, Ramos I, Hai R, Schmolke M, García-Sastre A, Fernandez-Sesma A, Palese P (2011). The influenza virus protein PB1–F2 inhibits the induction of type I interferon at the level of the MAVS. adaptor protein. PLoS Pathog.

[42] Gadotti AC, de Castro Deus M, Telles JP, Wind R, Goes M, Ossoski RG, de Padua AM, de Noronha L, Moreno-Amaral A, Baena CP, Tuon FF (2020). IFN–γ is an independent risk factor associated with mortality in patients with moderate and severe COVID-19 infection. Virus Res.

[43] Nathan CF, Murray HW, Wiebe ME, Rubin BY (1983). Identification of interferon–gamma as the lymphokine that activates human macrophage oxidative metabolism and antimicrobial activity. J Exp Med.

[44] Velu PP, Lucas CD, Conway MA (2021). Post–mortem dissection of COVID-19: a pathogenic role for macrophages?. Intensive Care Med.

[45] Olbei M, Hautefort I, Modos D, Treveil A, Poletti M, Gul L, Shannon-Lowe CD, Korcsmaros T (2021). SARS–CoV–2 causes a different cytokine response compared to other cytokine storm–causing respiratory viruses in severely ill patients. Front Immunol.

[46] Wilson JG, Simpson LJ, Ferreira AM, Rustagi A, Roque J, Asuni A, Ranganath T, Grant PM, Subramanian A, Rosenberg-Hasson Y, Maecker HT (2020). Cytokine profile in plasma of severe COVID-19 does not differ from ARDS and sepsis. JCI Insight..

[47] Jablonska E, Jablonski J (2002). Effect of IL-18 on the release of IL–6 and its soluble receptors: sIL–6R α and sgp130 by human neutrophils. Immunol Invest.

[48] Slaats J, Ten Oever J, van de Veerdonk FL, Netea MG (2016). IL–1β/IL–6/CRP and IL-18/ferritin: distinct inflammatory programs in infections. PLoS Pathog.

[49] Van Den Munckhof I, ter Horst R, Schraa K, Stienstra R, de Graaf J, Riksen N, Joosten L, Netea M, Rutten J (2019). IL-18 binding protein: a novel biomarker in obesity-related atherosclerosis that modulates lipoprotein metabolism. Atherosclerosis.

[50] Canna SW, Girard C, Malle L, de Jesus A, Romberg N, Kelsen J, Surrey LF, Russo P, Sleight A, Schiffrin E, Gabay C (2017). Life–threatening NLRC4–associated hyperinflammation successfully treated with IL-18 inhibition. J Allergy Clin Immunol.

[51] Safety, Tolerability, and Treatment Effect of Belnacasan in Patients With COVID-19—Full Text View—ClinicalTrials.gov [Internet]. Clinicaltrials.gov. 2022 [cited 6 May 2022]. Available from: https://clinicaltrials.gov/ct2/show/NCT05164120?id=NCT05164120&draw=2&rank=1&load=cart

[52] Wu G, Zhu Q, Zeng J, Gu X, Miao Y, Xu W, Lv T, Song Y (2019). Extracellular mitochondrial DNA promote NLRP3 inflammasome activation and induce acute lung injury through TLR9 and NF–κB. J Thorac Dis.

[53] Gu Y, Kuida K, Tsutsui H, Ku G, Hsiao K, Fleming MA, Hayashi N, Higashino K, Okamura H, Nakanishi K, Kurimoto M (1997). Activation of interferon-γ inducing factor mediated by interleukin-1β converting enzyme. Science.

[54] Honore PM, Spapen HD (2018). What a clinician should know about a renal replacement membrane?. J Transl Internal Med.

[55] Atan R, Crosbie DC, Bellomo R (2013). Techniques of extracorporeal cytokine removal: a systematic review of human studies. Ren Fail.

