# Supplementary material for: Elevated free interleukin-18 associated with severity and mortality in prospective cohort study of 206 hospitalised COVID-19 patients
Source: Intensive Care Med Exp. 2023 Feb 24;11:9. doi: 10.1186/s40635-022-00488-x (PMC9949911; doi:10.1186/s40635-022-00488-x)
Supplement: Supplementary file 1 — Additional file 1. Supplementary Methods. Supplementary Results. [file 40635_2022_488_MOESM1_ESM.docx]

SUPPLEMENT

Elevated Free Interleukin–18 Associated with Severity and Mortality in Prospective Cohort Study of 206 Hospitalised COVID–19 Patients

Dr Syed M T Nasser,^1,2^ Dr Anas A Rana,^3^ Dr Rainer Doffinger,^4^ Dr Andreas Kafizas,^5,6^ Dr Tauseef A Khan,^7^ Dr Shuaib Nasser^8^

^1^Intensive Care Department, Surrey and Sussex NHS Trust, UK; ^2^Intensive Care Department, Royal Surrey County Hospital, UK (current); ^3^Centre for Computational Biology, Birmingham University, UK; ^4^Department of Clinical Biochemistry and Immunology, Cambridge University Hospitals NHS Trust, UK; ^5^The Grantham Institute for Climate Change and the Environment, Imperial College London, South Kensington, London, UK; ^6^Department of Chemistry, Molecular Science Research Hub, Imperial College London, White City, London, UK; ^7^Department of Nutritional Sciences, Faculty of Medicine, University of Toronto, Canada; ^8^Department of Allergy, Cambridge University Hospitals NHS Trust, UK

***Corresponding Author Details***: Dr Syed M T Nasser MBBS ([syed.nasser1@nhs.net](mailto:syed.nasser1@nhs.net)); Intensive Care Department, Royal Surrey County Hospital NHS Foundation Trust; Egerton Road, Guildford GU2 7XX; Tel.: +44 1483 57122. Ext: 2177

2

# Contents

[Contents](#_bookmark0) 2

[Supplementary Methods](#_bookmark1) 3

[Law of Mass Action Calculation for Free IL-18 (fIL-18)](#_bookmark2) 3

[Supplementary Results](#_bookmark7) 4

Supplementary Figure 1 5

Supplementary Table 1 6

Supplementary Figure 2 8

[References 9](#_TOC_250000)

3

## Supplementary Methods

Law of Mass Action Calculation for Free IL-18 (fIL-18)

The free IL-18 concentration was not directly measured, but was derived from the IL-18 binding protein concentration and total IL-18 concentration, in accordance with the law of mass action:[1](#_bookmark8)

*K* = *CR* × *CL*

*D*

*CRL*

(Equation 1)

where KD is the dissociation constant for the equilibrium, CR is the concentration of the free receptor (total IL-18 binding protein), CL is the concentration of the free ligand (total IL-18) and CRL is the concentration of the complex between the receptor and ligand. As only the original concentrations of CR and CL are known, the concentrations are given by:

(Equation 2a) and

(Equation 2b)

*CR* = *C*0,*R* − *CRL*

*CL* = *C*0,*L* − *CRL*

where C0,R and C0,L are the original concentrations of CR and CL, respectively. Rearranging these equations,[2](#_bookmark9) the concentration of the complex, for the general case of 1:1 ligand-receptor interaction, is:

((*C*0,*R* + *C*0,*L* + *KD*) − (*C*0,*R* + *C*0,*L* + *KD*)2 − (4 × *C*0,*R* × *C*0,*L* ))

(Equation 3)

*CRL* = 2

and therefore, the concentration of the ligand, for the general case of 1:1 ligand-receptor interaction, is:

((*C*0,*L* − *C*0,*R* − *KD*) − (*C*0,*R* + *C*0,*L* + *KD*)2 − (4 × *C*0,*R* × *C*0,*L* ))

(Equation 4)

*CL* = 2

Concentrations of the total IL-18 were measured using Human total IL-18/IL-1F4 Quantikine ELISA kit (R&D Systems, 5 Minneapolis, MN, USA) and IL-18bp concentrations were measured using Human IL-18 BPa Quantikine ELISA kit (R&D Systems, Minneapolis, MN, USA) with values given in pg.ml-1. These were converted into molarities using masses of 24 and 18 kDa for the IL-18 binding protein and IL-18, respectively.[3](#_bookmark10) The free IL-18 concentration was calculated using Equation 4 using a KD of 0.05.[4](#_bookmark11)

4

## Supplementary Results

Recent work found that the Kd of IL-18 to IL-18bp was between 0.03 - 0.05 nM, as against 0.4 nM used historically for the calculation of fIL-18.4 We have chosen to present data utilising 0.05 nM value primarily, given the recent evidence showing its greater accuracy, but to also present fIL-18 values calculated with a Kd =

0.4 nM, for IL-18 parameter profiles by mortality outcome in the full cohort (Supplementary Figure 1) and for all regression analyses (Supplementary Table 1).

Additionally, IL–18 parameter profiles using a Kd = 0.05 nM are presented for the ventilated cohort (those who, at any point during admission, were artificially mechanically ventilated): Supplementary Figure 2.

5

5


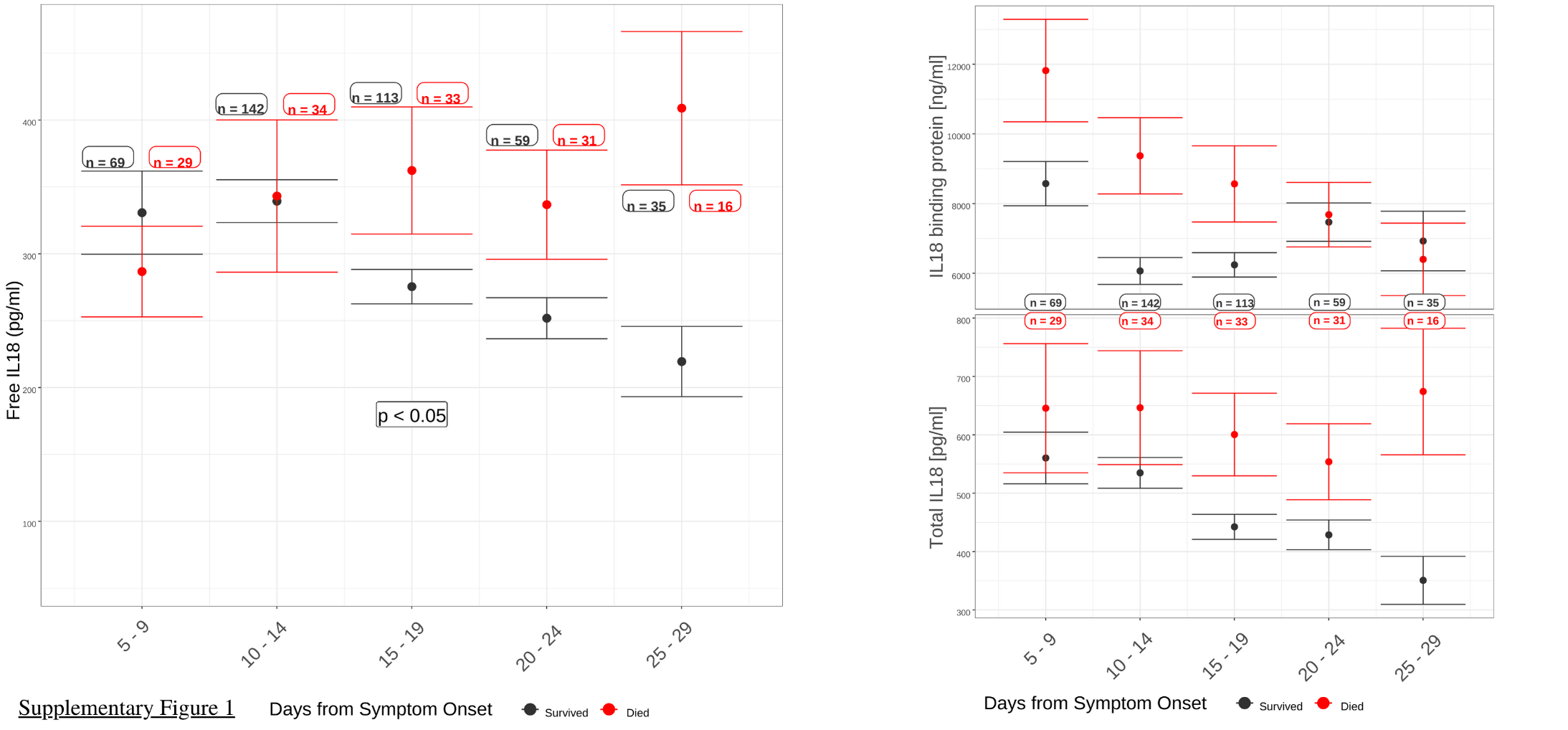


**Supplementary Figure 1: IL–18 parameter profiles by day from symptom onset (Kd = 0.4 nM). a)** Levels of fIL–18 increase between days 1–14 days of symptoms in 206 COVID-19 positive patients from baseline. From day 15 onwards, fIL–18 levels diverge between survival and 60-day mortality groups (p < 0.05). Mean fIL–18 level in 442 healthy, male volunteers is 175 pg/ml (not shown). **b)** Levels of IL-18 binding protein (IL-18bp) after symptom day 20 overlap between mortality groups. **c)** Total IL–18 increases in 60–day non–survivors from symptom day 15 onwards. Error bars represent standard error of the mean. *Survivors (black): 499 samples from 165 patients. Non–survivors (red)*: *162 samples from 41 patients.*

6

| Supplementary Table 1: Regression analysis for primary and secondary outcomes from symptom day 15 onwards; K_d_ = 0.4 nM | | | |
| --- | --- | --- | --- |
| **Outcome**  K_d_ = 0.4 nM | Highest Free IL–18 response coefficient (standard error) | Odds Ratio per 50 pg/ml increase in highest free IL–18 (95% confidence interval) | Significance |
| **Primary Outcome** |  |  |  |
| Pa02/Fi02 Ratio (mmHg) | – 0.594  (0.22) |  | **p < 0.01** |
| **Secondary Outcomes** |  |  |  |
| *Mortality* |  |  |  |
| Death at 60 days from symptom onset (crude mortality) |  | 1.17  (1.02 – 1.37) | **p < 0.05** |
| Death at 60 days from symptom onset (hypoxaemic respiratory failure) ✸ |  | 1.31  (1.11 – 1.60) | **p < 0.01** |
| *Organ–support Dependency* |  |  |  |
| Any organ support ✦ |  | 1.18  (1.03 – 1.37) | **p < 0.03** |
| Artificial mechanical ventilation only |  | 1.21  (0.94 – 1.66) | p > 0.1 |
| Per numbers of organs supported ✦ | 110.05  (45.39) |  | **p < 0.03** |
| Per numbers of organs supported (hypoxaemic respiratory failure) ✦ ✸ | 141.12  (42.15) |  | **p < 0.01** |
| *Biochemical Inflammatory Markers* |  |  |  |
| C–reactive Protein (CRP) | 0.68  (0.50) |  | p > 0.1 |
| Lymphocyte (as % of white blood cells) | – 88.41  (219.71) |  | p > 0.5 |
| Neutrophil / Lymphocyte ratio | 2.04  (2.80) |  | p > 0.3 |
| Neutrophil / Lymphocyte ratio (hypoxaemic respiratory failure) ✸ | 2.77  (2.73) |  | p > 0.3 |

✸ The “hypoxaemic respiratory failure group” excludes 4 patients whose calculated Pa02/Fi02 ratio from values recorded in the last 24 hours of life did not fall below 300 mmHg at any time. The group is used to exclude those who died from causes other than hypoxaemic respiratory failure.

- “Organs supported” refers to any of: vasopressor support; artificial mechanical ventilation; renal replacement therapy

8


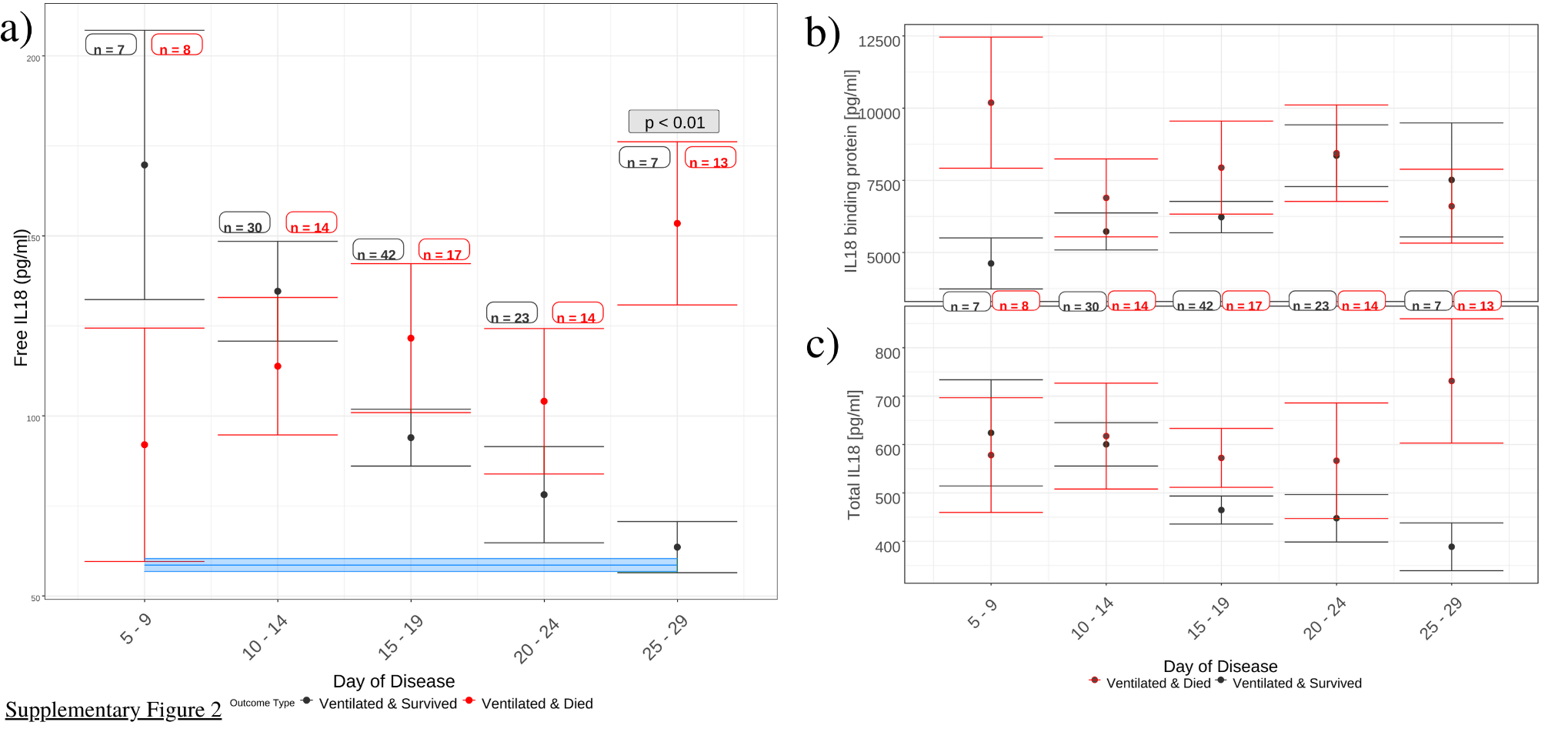


**Supplementary Figure 2: IL–18 parameter profiles by day from symptom onset (Kd = 0.05 nM) in ventilated cohort. a)** Free IL18 (fIL-18 / pgml^-1^) levels in the ventilated-survived cohort show a divergent trajectory to the ventilated-died cohort. Survivors show higher mean fIL-18 than non-survivors (days 5-9; p = 0.07). By days 25-29 however, divergence in levels of fIL-18 is large and significant (63.6 pg/ml [survived] vs 153.5 pg/ml [died]; p < 0.01). **b)** High IL-18 binding protein (IL–18bp) levels early in the disease are the cause of low fIL-18 levels in the mortality group. **c)** Levels of Total IL-18 overlap until time point day 24, thereafter diverging, giving a high fIL-18 value at days 25-29 in the ventilated-died group. Rising levels of IL-18bp and falling levels of Total IL-18 throughout the disease course in the survival group, underpin their steady fall in fIL-18 from day 5 to day 29. *Error bars represent standard error of the mean. Ventilated and survived: (black): 156 samples; 19 patients; Ventilated and died (red): 78 samples; 12 patients*

9

## References

1. Kenakin T. The mass action equation in pharmacology. British journal of clinical pharmacology. 2016 Jan;81(1):41-51.
2. Hulme EC, Trevethick MA. Ligand binding assays at equilibrium: validation and interpretation. British journal of pharmacology. 2010 Nov;161(6):1219-37.
3. Gaggero A, De Ambrosis A, Mezzanzanica D, Piazza T, Rubartelli A, Figini M, Canevari S, Ferrini S. A novel isoform of pro- interleukin-18 expressed in ovarian tumors is resistant to caspase-1 and-4 processing. Oncogene. 2004 Sep;23(45):7552-60.
4. Girard C, Rech J, Brown M, Allali D, Roux-Lombard P, Spertini F, Schiffrin EJ, Schett G, Manger B, Bas S, Del Val G. Elevated serum levels of free interleukin-18 in adult-onset Still’s disease. Rheumatology. 2016 Sep 10;55(12):2237-47.
